# Ferroptosis as a novel targeted therapy in gynecological malignant tumors

**DOI:** 10.1007/s12672-026-04686-x

**Published:** 2026-03-13

**Authors:** Congxiang Yu, Yuefei Li, Yangxin Fu, Ruizhe Yang, Zorica Nakevska, Pooja Praveen Kumar, An Nhien Tong, Nan Yang, Dan Liu, Chao Chen, Ling Ouyang, Zhijun Xia

**Affiliations:** 1 Department of Obstetrics and Gynecology, Affiliated Hospital of Inner Mongolia Medical University, Hohhot, 010050 Inner Mongolia China; 2 Shengjing Hospital of China Medical University, Shenyang, 110122 Liaoning China; 3 Department of Obstetrics and Gynecology, Inner Mongolia Maternal and Child Health Care Hospital, Hohhot, 010020 Inner Mongolia China; 4 Department of Oncology, Faculty of Medicine and Dentistry, University of Alberta, Edmonton, AB Canada; 5 Department of Cellular Biology, Faculty of Medicine and Dentistry, University of Alberta, Edmonton, AB Canada; 6 The Fourth Affiliated Hospital of China Medical University, Shenyang, 110122 Liaoning China; 7 Department of Gynaecology and Obstetrics, General Hospital of Northern Theater Command, Shenyang, 110122 Liaoning China; 8 Obstetrics and Gynecology Hospital of Fudan University, Shanghai, 60513996 China

**Keywords:** Gynecological, Malignant tumors, Ferroptosis, Cervical carcinoma, Endometrial carcinoma, Ovarian carcinoma, Glutathione peroxidase 4

## Abstract

Ferroptosis, an emerging form of regulated cell death driven by iron-dependent lipid peroxidation, represents a promising therapeutic target in oncology. Gynecological malignancies—cervical, endometrial, and ovarian cancers—are frequently associated with therapeutic resistance due to defective cell death regulation. This review delineates cancer-specific ferroptosis regulatory networks across these malignancies. In cervical cancer, high-risk HPV oncoproteins (E6/E7) rewire oxidative stress and lipid metabolism, leading to stage-dependent ferroptosis vulnerability and supporting combination approaches with immunotherapy. In endometrial cancer, ELK1-mediated GPX4 upregulation drives chemoresistance, highlighting ferroptosis induction as a strategy to overcome treatment failure. In ovarian cancer, iron overload promotes metastasis, while p53, lipid-modifying enzymes (SCD1/FADS2), and the tumor microenvironment (e.g., CXCL8/CXCR2 axis) modulate ferroptosis sensitivity, providing avenues to target aggressive subtypes such as clear cell carcinoma. We conclude that targeting ferroptosis offers a transformative strategy for gynecological cancers.

Gynecological malignancies, including cervical, endometrial, and ovarian carcinomas, represent a major global health burden due to their high prevalence and mortality [1]. Like many cancers, their progression and resistance to therapy are frequently linked to dysregulated cell death pathways. Cell death plays a vital role in normal development, homeostasis, and the prevention of hyperproliferative diseases, including cancer [2].

Ferroptosis, a distinct type of cell death characterized by iron-dependent lipid peroxidation, was first identified by Dixon in 2012. This form of cell death is unique from apoptosis, necrosis, and autophagy, differing in its morphological, biochemical, and genetic characteristics. Dixon’s research revealed that the cell death pathway activated by the oncogenic RAS-selective small molecule erastin differs from thepathways commonly involved in programmed cell death. Erastin induces ferroptosis in cancer cells through the accumulation of deferoxamine (DFO)-sensitive reactive oxygen species (ROS), even in cells lacking a functional mitochondrial electron transport chain (ETC). This process is regulated by various cellular components, including iron metabolism [3], lipid metabolism [4], and antioxidant defenses, particularly through the glutathione (GSH) and glutathione peroxidase 4 (GPX4) pathways [5]. Meanwhile, RSL3 can initiate ferroptosis by directly inhibits the enzyme GPX4, thereby preventing the detoxification of lipid peroxides.The subsequent iron-dependent accumulation of reactive oxygen species (ROS) ultimately triggers ferroptosis [6].These mechanisms of regulating ferroptosis mentioned above are currently a significant area of research interest (Fig. 1).

**Fig. 1:**
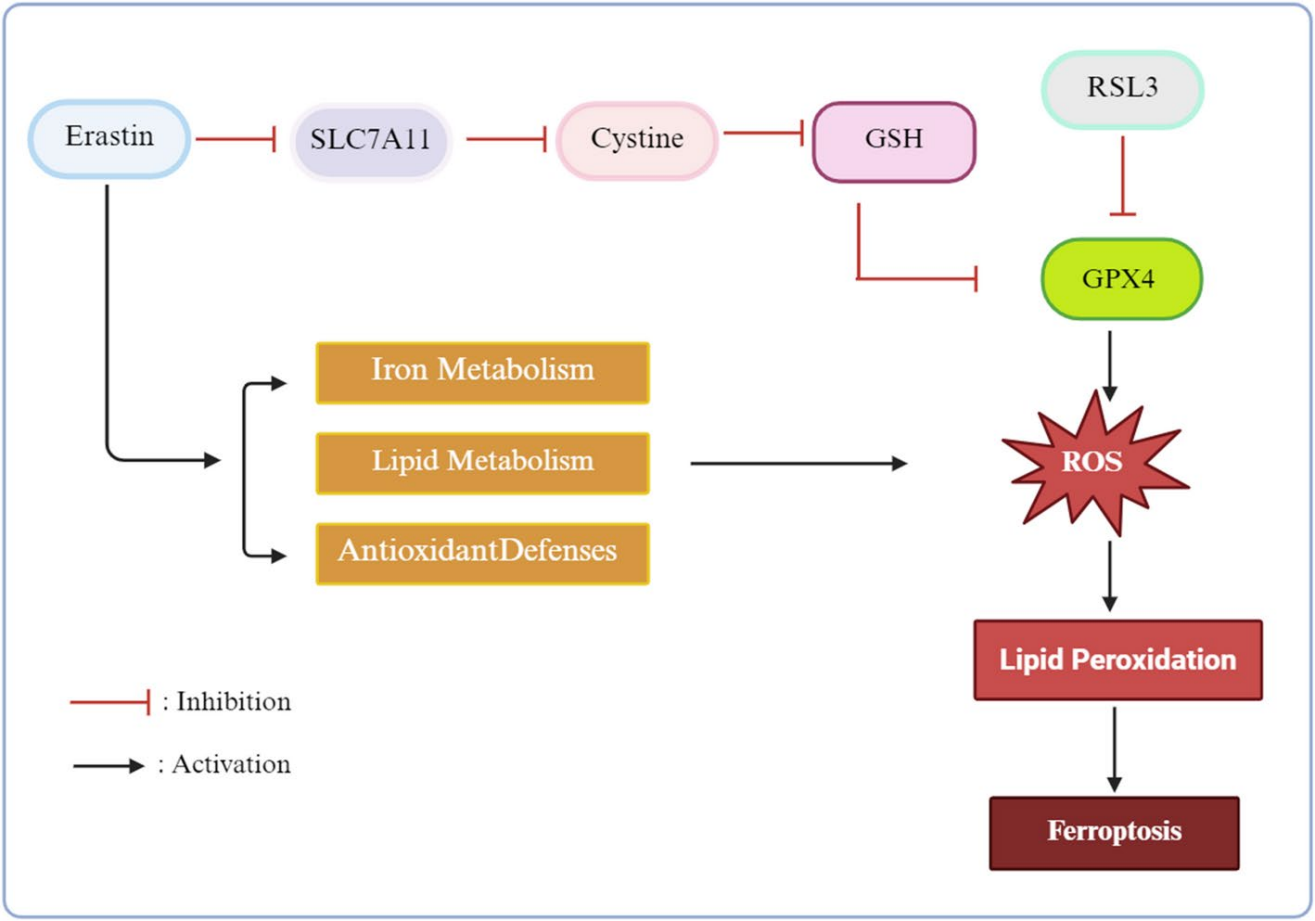
Molecular Mechanisms of Ferroptosis Induction by Erastin and RSL3. Erastin induces ferroptosis through the coordinated dysregulation of iron metabolism, lipid metabolism, and antioxidant defenses. Particularly by inhibiting SLC7A11, a core subunit of the system Xc⁻ transporter, thereby blocking cellular cystine uptake. Since cystine is essential for cysteine synthesis-a critical precursor of GSH-this inhibition depletes intracellular GSH levels.The loss of GSH inactivates GPX4, leading to the accumulation of lipid ROS and ultimately triggering ferroptosis. RSL3 directly inhibits the enzymatic activity of GPX4, impairing the detoxification of lipid peroxides. This results in iron-dependent accumulation of ROS, which also culminates in ferroptosis

Ferroptosis has been linked to pathological tissue damage caused by above chemotherapy drugs and ischemia/reperfusion events [7–10]. Iron, predominantly stored in ferritin, is integral to proteins that compose hemoglobin and [Fe-S] clusters. These iron-containing proteins are essential for various cellular functions such as energy metabolism, ATP synthesis, oxygen transport, DNA synthesis and repair, making adequate iron levels crucial for cells [11–12]. In mammalian cells, iron is distributed among mitochondria (approximately 16 µM), cytosol (approximately 6 µM), nuclei (approximately 7 µM), and lysosomes (approximately 16 µM) [13]. The cellular labile iron pool (LIP), consisting of redox-active iron (Fe^2+^), represents a very small fraction of free iron. Despite its minimalquantity, this pool can significantly interfere with numerous cellular functions, given that iron homeostasis is vital for many organisms. It plays a crucial role in processes such as ferroptosis induction, cell division, and proliferation. Under physiological conditions, the liver monitors the body’s systemic iron levels and dynamically produces the hormone hepcidin, which is a central regulator of iron homeostasis [14]. In recent years, hepcidin has become a focal point as a potential therapeutic target for iron-related disorders such as anemia and iron overload [15]. Redox-active iron generates ROS, which can damage lipids, proteins, and DNA. These damaged molecules can induce senescence or cell death, leading to a variety of diseases [16]. Research has shown that cancer cells have a higher iron requirement for survival compared to normal cells [17]. Alvarez et al. demonstrated that increasing intracellular LIP by suppressing cysteine desulfurase(NFS1)sensitizes lung cancer cells to ferroptosis and reduces tumor growth in vivo [18]. However, it has been shown that injections of iron preparations can cause serious side effects, such as the development of sarcomas, and can exacerbate diseases. Consequently, understanding and targeting ferroptosis has emerged as a promising therapeutic strategy in cancer treatment [19]. Although many of the mechanisms involved in ferroptosis were identified in past decades, the specific contributions of ferroptosis in gynecological malignancies remain largely unknown. In this review, we explore the potential of modulating ferroptosis to overcome resistance to cancer therapies. We aim to provide insights and references that will guide further investigation into this exciting area.

## Cervical cancer

In recent years, cervical cancer has consistently ranked among the top three cancers affecting middle-aged women worldwide, and it remains the most prevalent cancer in low-resource countries. In 2018, approximately 570,000 women were diagnosed with cervical cancer, and 311,000 succumbed to the disease [20]. Cervical squamous cell carcinoma, the predominant form of this cancer, is closely linked to human papillomavirus (HPV) infection. Despite aggressive efforts to promote the HPV vaccine and advances in screening technologies, cervical cancer continues to be a leading cause of death among women in underdeveloped regions [21]. Therefore, it is crucial to identify more effective and safer therapeutic targets and treatments for cervical cancer.

The role of ferroptosis in cervical cancer represents a burgeoning field of interest, particularly in its potential to enhance the efficacy of chemotherapeutic agents. Research is increasingly focusing on the interaction between HPV oncogenes and ferroptosis regulation, providing insights into the unique vulnerabilities of cervical cancer cells. HPV proteins promote lipid modification and oxidative stress, factors that contribute to high-risk human papillomavirus (HR-HPV) infection and their persistence against immune system clearance. This contributes to a cascade of cellular changes, including increased cell proliferation, evasion of cell death, genomic instability, and ultimately, the development of cervical cancer [22]. Wang et al. discovered that cervical tissue infected with HR-HPV exhibited heightened sensitivity to ferroptosis during the squamous intraepithelial lesion (SIL) stage [23]. However, cervical squamous cell carcinoma (SCC) tissues and cell lines showed resistance to ferroptosis in response to its persistent induction. It is hypothesized that metabolic reprogramming serves as a critical mechanism for cervical cancer cells to overcome ferroptosis.Notably, cervical lesions that exhibit anti-ferroptotic effects have been found to upregulate KRAS, thereby promoting tumorigenesis in SCC. This evolving understanding underscores the complexity of ferroptosis’s role in SCC and highlights its potential as a therapeutic target.

The viral E6 and E7 oncogenes are central to the oncogenic potential of HPV. E6 induces mitochondrial proton leak, which elevates ROS levels both within mitochondria and throughout the whole cells, thereby driving the oxidative stress, DNA damage and genomic instability, thereby promoting carcinogenesis [24]. Furthermore, E6 stabilizes hypoxia-inducible factor 1α (HIF-1α) by blocking its VHL-mediated degradation.This increases the expression of glucose metabolism-related HIF-1-driven genes, supplying the energy and metabolic intermediates required for the growth of tumors [25]. Separately, E6 binds to E6 AP, leading to the degradation of p53. Loss of p53 function may subsequently enhance SLC7A11 activity, contributing further to elevated intracellular ROS levels [23]. Additionally, the E6/E7 oncoproteins activate the PI3K/Akt/mTOR pathway, which markedly upregulates key enzymes involved in lipid synthesis-including ATP-citrate lyase (ACLY), fatty acid synthase (FASN), and stearoyl-CoA desaturase 1(SCD1)-thereby stimulating lipid synthesis [26]. Furthermore, E7 elevates ROS levels by driving a dysregulated increase in mitochondrial activity without adequate quality control, thereby establishing a cellular microenvironment highly conducive to malignant transformation. [27–28] Inhibiting the expression of E6 and E7 oncogenes in HPV-positive cancer cells leads to rapid induction of cellular senescence under hypoxic conditions. However, this senescence can be reversed by experimentally reactivating mTOR signaling [29], highlighting a key escape mechanism from a major tumor-suppressive defense, which allows for persistent infection and the development of cervical tumors. Furthermore, it has been reported that head and neck squamous cell carcinoma (HNSCC) cells expressing the E6 and E7 oncoproteins from HPV16 are sensitive to erastin-induced ferroptosis due to reduced levels of GPX4 and GSH [30]. GSH plays a crucial role in regulating ferroptosis and serves as a critical component for the antioxidant GPX4, which prevents the accumulation of lipid ROS [31–32]. The inactivation of GPX4 leads to the continuous accumulation of ROS on membrane lipids, causing cellular lipid peroxidation and triggering ferroptosis [33].

**Fig. 2:**
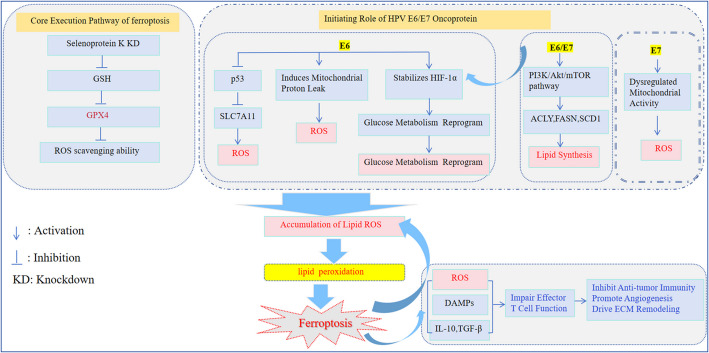
Molecular Mechanisms of HPV-Induced Ferroptosis Regulation. E6/E7 induce mitochondrial proton leak and elevate ROS, driving oxidative stress and genomic instability; E6 may promote SLC7A11 function via p53 degradation; E6/E7 activate the PI3K/Akt/mTOR pathway, upregulating lipid synthesis enzymes (ACLY, FASN, SCD1). HPV16-positive head and neck squamous cell carcinoma cells show sensitivity to erastin due to reduced GPX4 and GSH levels; knockdown of Selenoprotein K decreases GPX4/GSH, impairs ROS scavenging, and exacerbates ferroptosis in HeLa cells Ferroptosis generates DAMPs and immunosuppressive cytokines (IL-10, TGF-β), impairing effecter T-cell function. Ultimately inhibit anti-tumor immunity, promote angiogenesis, and drive ECM remodeling

Additionally, studies have demonstrated that knockdown of the Selenoprotein K gene leads to significant decreases in GPX4/GSH levels, impaired ROS scavenging ability, and exacerbated ferroptosis in HeLa cells, potentially reducing cervical tumor size by regulating ferroptosis [34]. Ferroptosis also induces the production of damage-associated molecular patterns (DAMPs) [35] and reactive ROS, along with the release of immunosuppressive cytokines including interleukin-10(IL-10) and transforming growth factor-beta(TGF-β) [36]. These effects collectively impair effector T cell function and ultimately favor processes that inhibit anti-tumor immunity, promote angiogenesis, and drive extracellular matrix(ECM) remodeling.Given this context, research demonstrates that combining Vascular Endothelial Growth Factor A(VEGFA) inhibitors with immune checkpoint blockers (e.g., anti-PD-1/PD-L1) can rejuvenate T-cell antitumor activity and improve therapeutic outcomes [37]. Thus, combining immunotherapy with ferroptosis inducers emerges as a promising novel strategy for cervical carcinoma, particularly in HPV-positive patients. It may promote the elimination of HR-HPV infection and the reversal of SIL by activating ferroptosis, potentially preventing the development of cervical cancer. GPX4 may be a potential molecular target, although further in-depth research is required to confirm this (Fig. 2). 

## Endometrial cancer

Endometrial cancer (EC) is the most commonly encountered malignant epithelial tumor of the uterus. It is characterized by a variety of architectural patterns such as glandular, papillary, or solid structures, and typically arises from endometrioid differentiation in a background of atypical hyperplasia [37].The morbidity and mortality associated with EC are on the rise [38], with the five-year survival rate for patients diagnosed with advanced disease being less than 20%, even with combination therapy [39–41]. This low survival rate is often attributed to resistance to chemotherapeutic drugs [42], emphasizing the need for studies aimed at revealing molecular resistance mechanisms and identifying novel therapeutic targets [43]. Current treatment strategies for EC include immune checkpoint inhibitors [44–45], DNA damage response inhibitors [46], and inhibitors of specific cellular pathways [47]. However, the addition of metformin to these therapeutic combinations has not shown improved outcomes [48]. There remains a significant challenge in predicting EC prognosis and identifying convenient and effective biomarkers.

Recent studies suggest that EC exhibits altered iron metabolism and oxidative stress pathways, indicating potential susceptibility to ferroptosis, a regulated form of cell death influenced by various molecular signaling pathways through epigenetic, transcriptional, and post-translational mechanisms [49–50]. Iron plays a unique role in the female reproductive system and is closely associated with several gynecological diseases [51–52]. A previous study categorized EC samples into four clusters based on expression levels of 60 ferroptosis-related genes, such as prostaglandin-endoperoxide synthase 2 (PTGS2/COX2). These genes are all closely linked to the ferroptosis process, suggesting its significant role in the development and progression of EC. The study also noted considerable differences in the degree of infiltration of 21 types of immune cells across tumors, indicating heterogeneity in immune responses and the likely significant role of ferroptosis [53–54]. Further research identified that silencing PTPN18, a member of the epidermal growth factor receptor family, might induce ferroptosis by targeting the p-p38/GPX4/xCT axis, underscoring the critical role of ferroptosis in reducing EC cell growth and survival [55]. It was observed that in EC tissues and cell lines, GPX4 expression was upregulated. Knockdown of GPX4 led to increased Fe2+, malondialdehyde (MDA), and ROS levels, decreased mitochondrial membrane potential (MMP), and activated ferroptosis, which inhibited proliferation and migration of EC cells. The transcription factor ELK1 regulates GPX4 transcription by binding to its promoter, inhibiting ferroptosis, and promoting EC progression [56]. In GPX4 knockout mouse models, lipid peroxidation was observed, potentially activating ferroptosis and resulting in cell loss or lethality [57]. Another study showed that in EC tissues and Ishikawa cell lines, expression of Fancd2, a key player in genomic maintenance in response to DNA damage, was upregulated and correlated with sensitivity to chemotherapeutic agents. Knockdown of Fancd2 increased ROS, GSH, and Fe2 + levels and MDA activity, while decreasing SLC7A11 and GPX4 expression in Ishikawa/TAX cells, indicating that ferroptosis was activated [58–59]. These findings highlight the potential of targeting and regulating of GPX4-induced ferroptosis pathway to overcome chemotherapeutic resistance in EC.

## Ovarian cancer

Ovarian cancer (OC) ranks as the seventh most common cancer among women worldwide and the fifth leading cause of cancer-associated deaths [60]. This high mortality rate is largely due to late diagnoses [61], the cancer’s propensity for drug resistance [62], and frequent recurrence [63]. Among ovarian cancers, epithelial ovarian cancer (EOC) accounts for 50–70% of cases and is the fifth most deadly cancer of the female reproductive system. The American Cancer Society estimates that there will be 19,710 new cases and 13,270 deaths from EOC in the United States in 2023 [64]. EOC typically originates from the lining of the fallopian tubes or the surface layer of the ovaries. The lack of distinctive symptoms in the early stages (stages I and II) contributes to the disease often being advanced at diagnosis with 76% of patients presenting with intra-abdominal implants, hepatic parenchymal, thoracic, and intracranial metastases (stages III and IV). Despite advances in cancer treatments, including the introduction of immunotherapy and targeted therapies, these have yet to show substantial efficacy [65–70]. Moreover, recent studies suggest an emerging trend of EOC manifesting at younger ages.

Research indicates that iron plays a significant role in the metastatic spread of ovarian cancer by enhancing the production of interleukin 6 (IL-6), a cytokine that promotes tumor angiogenesis and inflammation [71–72]. An increase in iron levels within ovarian cancer cells has been shown to decrease the reducibility of lipid peroxides, whereas a reduction in iron levels has the opposite effect. Knocking down GPX4 in ovarian cancer cells reduces intracellular iron and levels of IL-6 and TNF-α, suggesting that diminished iron levels may curb proinflammatory cytokine production and lipid peroxide reducibility [73]. It has also been discovered that in both high-grade serous ovarian cancer tissues and ovarian cancer tumor-initiating cells (TICs), the transferrin receptor 1 (TFRI) is overexpressed, and iron efflux pump expression is reduced, thereby increasing intracellular free iron content. Conversely, reducing intracellular iron content can inhibit ovarian cancer cell proliferation and intraperitoneal dissemination, highlighting iron’s crucial role in the development, invasion, and metastasis of ovarian cancer cells [74]. In an ex vivo culture of organoids derived from EOC patients and an in vivo study of peritoneal metastases in animals, it was found that taking advantage of stearoyl-CoA desaturase-1(SCD1) and acyl-CoA 6-desaturase (FADS2) inhibition can synergistically abrupt ROS production to induce ferroptosis, sensitize cells to cisplatin-mediated cytotoxicity in vitro, and induce EMT suppression [75]. In parallel, it was found that tumor-associated macrophages promote endothelial cell resistance to ferroptosis through the CXCL8/CXCR2/NF-κB axis, which upregulates the ferroptosis-related proteins SLC7A11 and GPX4 in EOC cells [76]. Conversely, p53 promotes ferroptosis by directly inhibiting SLC7A11 expression. This inhibition restricts L-cysteine uptake, which reduces GSH and GPX4 activity [77]. Additionally, p53 enhances cellular iron uptake by upregulating TFR1, thereby increasing the labile iron pool [78]. The amplified iron load promotes Fenton reaction, resulting in the initiation of lipid peroxidation. These p53-mediated effects collectively create a cellular environment permissive to ferroptosis.

Ovarian clear cell carcinoma (OCCC), recognized as one of the most aggressive forms of ovarian cancer, demonstrates greater resistance to chemotherapeutic treatments, underscoring the urgent need for effective chemotherapy options [79–80]. Human mammary epithelial (HME) cells may resist ferroptosis by maintaining intracellular cystine or glutathione levels [81]. Studies showed that EGFR mutant HME cells treated with auranofin, an inhibitor of the thioredoxin reductase/thioredoxin (TRX) system [82], and simultaneous glutathione depletion, led to reduced cell viability and induced lipid ROS [83–84]. Thus, these EGFR mutant cells oxidize less glutathione during cystine deprivation, allowing ROS accumulation and suggesting that intracellular cysteine plays a significant role in counteracting ROS and ferroptosis. Further research by Wisna et al. demonstrated that cysteine deprivation induces oxidative stress in glycolytic OCCC, disrupts Fe-S cluster synthesis and the mitochondrial electron transport chain, leading to ROS-induced ferroptosis and subsequent inhibition of OCCC tumor growth [85]. Additionally, treatment with the ferroptosis inducer erastin, both in vitro and in vivo, resulted in significant ferroptosis of cancer cells, decreased OCCC tumor growth, and improved survival in model mice [86]. These findings underscore the potential of targeting the ferroptosis pathway as a therapeutic strategy in the treatment of OCCC.

In conclusion, ferroptosis presents a compelling and novel therapeutic paradigm for gynecological malignancies. This review elucidates how the dysregulation of iron homeostasis, lipid metabolism, and the GPX4 antioxidant system contributes to therapy resistance in cervical, endometrial, and ovarian cancers, while also revealing cancer-specific vulnerabilities: HPV-mediated metabolic reprogramming in cervical carcinoma, the ELK1/GPX4 axis in endometrial cancer, and iron overload coupled with p53 inactivation in ovarian cancer, respectively. However, the clinical translation of this strategy faces significant challenges, including a poorly understood molecular basis for resistance-encompassing adaptive lipid repair (e.g., via FSP1), altered iron handling, and alternative antioxidant pathways-as well as the highly context-dependent role of the tumor microenvironment. Future research must therefore prioritize deciphering these complex regulatory networks, developing biomarkers for patient stratification, and designing tumor-selective inducers and rational combination therapies to ultimately transform these mechanistic insights into improved clinical outcomes (Table 1).

**Table 1:**
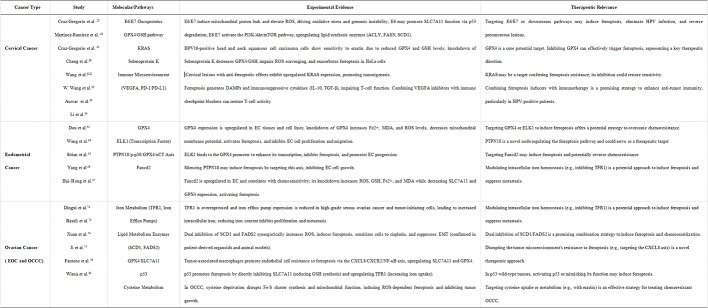
Summary key molecular targets, experimental evidence, and therapeutic relevance across gynecological cancers

## Data Availability

No datasets were generated or analysed during the current study.

## Abbreviations

DFO: Deferoxamine
ROS: Reactive oxygen species
ETC: Electron transport chain
GSH: Glutathione
GPX4: Glutathione peroxidase 4
LIP: Labile iron pool
HPV: Human papillomavirus
HR-HPV: High-risk human papillomavirus
SIL: Squamous intraepithelial lesion
SCC: Squamous cell carcinoma
HNSCC: Neck squamous cell carcinoma
EC: Endometrial cancer
PTGS2/COX2: Prostaglandin-endoperoxide synthase 2
MDA: Malondialdehyde
MMP: Mitochondrial membrane potential
OC: Ovarian cancer
EOC: Epithelial ovarian cancer
IL-6: Interleukin 6
TICs: Tumor-initiating cells
TME: Tumor microenvironment
TFRI: Transferrin receptor 1
SCD1: Stearoyl-CoA desaturase-1
FADS2: Acyl-CoA 6-desaturase
OCCC: Ovarian clear cell carcinoma
HME: Human mammary epithelial
TRX: Thioredoxin reductase/thioredoxin
DAMPs: Damage-associated molecular patterns
IL-10: Interleukin-10
TGF-β: Transforming growth factor-beta
